# Sensory and Choice Responses in MT Distinct from Motion Encoding

**DOI:** 10.1523/JNEUROSCI.0267-22.2023

**Published:** 2023-03-22

**Authors:** Aaron J. Levi, Yuan Zhao, Il Memming Park, Alexander C. Huk

**Affiliations:** ^1^Center for Perceptual Systems, Departments of Neuroscience and Psychology, The University of Texas at Austin, Austin, Texas 78705; ^2^Department of Neurobiology and Behavior, Stony Brook University, Stony Brook, New York 11794; ^3^Fuster Laboratory, University of California Los Angeles, Los Angeles CA 90095

**Keywords:** choice-related activity, decision-making, population decoding, temporal weighting, visual motion

## Abstract

The macaque middle temporal (MT) area is well known for its visual motion selectivity and relevance to motion perception, but the possibility of it also reflecting higher-level cognitive functions has largely been ignored. We tested for effects of task performance distinct from sensory encoding by manipulating subjects' temporal evidence-weighting strategy during a direction discrimination task while performing electrophysiological recordings from groups of MT neurons in rhesus macaques (one male, one female). This revealed multiple components of MT responses that were, surprisingly, not interpretable as behaviorally relevant modulations of motion encoding, or as bottom-up consequences of the readout of motion direction from MT. The time-varying motion-driven responses of MT were strongly affected by our strategic manipulation—but with time courses opposite the subjects' temporal weighting strategies. Furthermore, large choice-correlated signals were represented in population activity distinct from its motion responses, with multiple phases that lagged psychophysical readout and even continued after the stimulus (but which preceded motor responses). In summary, a novel experimental manipulation of strategy allowed us to control the time course of readout to challenge the correlation between sensory responses and choices, and population-level analyses of simultaneously recorded ensembles allowed us to identify strong signals that were so distinct from direction encoding that conventional, single-neuron-centric analyses could not have revealed or properly characterized them. Together, these approaches revealed multiple cognitive contributions to MT responses that are task related but not functionally relevant to encoding or decoding of motion for psychophysical direction discrimination, providing a new perspective on the assumed status of MT as a simple sensory area.

**SIGNIFICANCE STATEMENT** This study extends understanding of the middle temporal (MT) area beyond its representation of visual motion. Combining multineuron recordings, population-level analyses, and controlled manipulation of task strategy, we exposed signals that depended on changes in temporal weighting strategy, but did not manifest as feedforward effects on behavior. This was demonstrated by (1) an inverse relationship between temporal dynamics of behavioral readout and sensory encoding, (2) a choice-correlated signal that always lagged the stimulus time points most correlated with decisions, and (3) a distinct choice-correlated signal after the stimulus. These findings invite re-evaluation of MT for functions outside of its established sensory role and highlight the power of experimenter-controlled changes in temporal strategy, coupled with recording and analysis approaches that transcend the single-neuron perspective.

## Introduction

Primate middle temporal (MT) area plays a critical role in the perception of visual motion. A long line of study has established that MT's encoding of motion direction is quantitatively consistent with perceptual sensitivity, that noise in its responses is correlated with behavioral variability, and that causal perturbations of its activity affect motion perception in lawful and substantial ways (Newsome and Paré, 1988; Britten et al., 1992; Salzman et al., 1992; Britten et al., 1996). Owing to this powerfully integrated set of findings, many models and experiments have been able to assume that MT is the key place that the brain looks to for information about visual motion. However, these successes do not logically imply that MT only carries sensory information, leaving our understanding of MT at risk of overlooking additional signals and computations that are not aligned with representing motion for the sake of motion perception. In this work, we show that a novel manipulation of temporal strategy during motion discrimination reveals large signals in MT that are precisely related to components of performing the task, but neither of these sensory-related and choice-correlated signals directly impact psychophysical performance or reflect straightforward links between perceptual decisions and the sensory responses that informed them.

To directly test for and characterize nonsensory signals in MT, we manipulated the time course of psychophysical weighting while monkeys performed a direction-discrimination task, coupled with multineuron recordings analyzed via population-level approaches. We explicitly manipulated whether early or late parts of the stimulus had stronger or weaker motion evidence on average, which affected the time course of how subjects weighted the motion stimulus during task performance. This manipulation of the temporal weighting strategy caused a surprising and strong modulation of the sensory responses themselves that was not directly related to forming decisions about motion, and also provided critical leverage for “stress-testing” the relation between the time courses of decision formation and of choice-correlated activity.

When perceptual weighting was unconstrained, direction discrimination behavior was based primarily on early portions of the stimulus, and the sensory representation showed a standard and modest falloff over the course of stimulus presentation. When we shifted the temporal readout strategy to favor late portions of the stimulus, behavior relied preferentially on later stimulus epochs, but the sensory response did not change to match the time course of behavior. When subjects' temporal weighting strategy was then manipulated to preferentially rely on earlier portions of the stimulus, later portions of the sensory response were increased rather than decreased. The effects of this last condition were most striking, as a steep falloff in perceptual weighting over time was accompanied by an increase in late sensory-driven activity that led to a nonmonotonic time course of motion-driven response.

Choice-correlated activity during the stimulus was also altered by changes in the psychophysical weighting, and across these behavioral time courses, was always lagged relative to the periods when the subjects were “reading out” MT activity. The consistently decision-lagged signal negates a major role of feedforward sensory noise in the origin of choice-related signals in MT. Furthermore, this signal was not simple feedback linking a sensory response and a subsequent, corresponding decision, not just because the choice signals affected MT population activity differently than visual motion did; we also observed a distinct choice-related signal after stimulus offset that was linked to impending response, and which was distinct from simple premotor activity.

These multiple components of the MT response were lawful functions of the time course of decision formation and the anticipation of the response; however, they cannot be interpreted as either modulations of the encoding that play out in perceptual reports or as effects of readout mechanisms that would either correlationally (via feedforward mechanisms) or causally (via straightforward feedback mechanisms) align with the sensory response. Thus, there appear to be multiple, large components of MT activity that both affect its stimulus-driven response but are separable from it. These were evident even during a well studied direction discrimination task with tight control over motion readout strategy, expanding the conventional designation of MT as a simple, low-dimensional, sensory-encoding area.

## Materials and Methods

### Experimental design

#### Stimulus presentation and design.

Stimuli were presented using the Psychophysics Toolbox with MATLAB (MathWorks) using a DATAPixx I/O box (VPixx) for precise temporal registration (Eastman and Huk, 2012). Sample stimulus presentation code is available on request. Eye position was tracked using an Eyelink eye tracker (SR Research), sampled at 1 kHz. Monkey L was seated 57 cm away from a 150 cm × 86 cm rear-projection screen (IRUS; Draper) covering the central 106° × 73° of visual angle. Images were projected onto the screen by a PROPixx Projector (VPixx Technologies) driven at a resolution of 1920 × 1080 pixels at 120 Hz. Monkey N (M2) viewed stimuli on a 55 inch LCD (LG) display (resolution = 1920 × 1080 pixels; refresh rate = 60 Hz; background luminance = 26.49 cd/m^2^) that was corrected to have a linear γ function. M2 viewed the stimulus from a distance of 118 cm (such that the screen width subtended 54° of visual angle, and each pixel subtended 0.0282° of visual angle). Auditory feedback was played at the end of every trial, and fluid reward was delivered through a computer-controlled solenoid.

**Figure 1.**
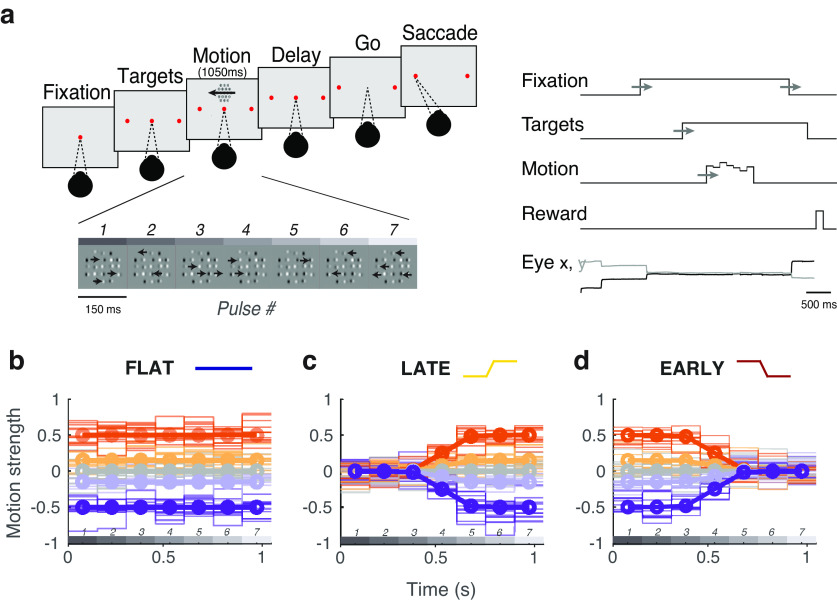
Sequence of trial events and manipulation temporal stimulus statistics. ***a***, Subjects fixated on a central point through the appearance of targets and motion stimulus until the disappearance of the fixation point (“go”). Choices were made with saccades to the target corresponding to the perceived net direction of motion. Initial fixation time, target-on duration, and time until fixation point disappearance were randomly varied. ***b–d***, Average stimulus strength per pulse (bold lines) and individual trial examples (semi-transparent lines) for trials of different strength and direction (denoted by sign). ***b***, In the flat stimulus, motion strength is constant over time on average. ***c***, In the late stimulus, motion strength is reduced on average in the first three pulses such that the highest motion expectation is late. ***d***, In the early stimulus, motion strength is reduced in the last three pulses such that the highest motion expectation is early. Motion pulse values in individual trials (semitransparent traces) vary considerably (for details, see Materials and Methods).

Subjects were required to discriminate the net direction of a motion stimulus and communicate their decision with an eye movement to one of two targets, placed on either side of the stimulus. The sequence of task events is presented in Figure 1*a*. A trial began with the appearance of a fixation point. Once the subject acquired fixation and held for 750–1300 ms, two targets appeared and remained visible until the end of the trial. Five hundred to one thousand milliseconds after target onset, the motion stimulus was presented at a range of eccentricities from 4° to 12° for a duration of 1050 ms. The fixation point was extinguished 500–1000 ms after motion offset, and the subject was then required to shift their gaze toward one of the two targets within 600 ms (saccade end points within 3° of the target location were accepted). All randomly varied stimulus events were drawn from a uniform distribution. If the animals ever moved their eyes outside of a 3° diameter window around the fixation point (before the fixation point disappeared), the trial was aborted. The timing of each event was randomly and independently jittered from trial to trial.

The reverse-correlation motion stimulus contained motion toward one direction or the opposite, with varying motion strength. Spatially, the stimulus consisted of a hexagonal grid of 19 Gabor elements, where individual Gabor elements were set to approximate the receptive field (RF) size of a V1 neuron, and the entire motion stimulus approximated the RF size of an MT neuron, which scaled based on eccentricity from fixation (Van Essen et al., 1981). Motion was presented by varying the phase of the sine-wave carrier of the Gabor elements. Each Gabor element underwent a sinusoidal contrast modulation over time with independent random phase. Gabor spatial frequency (0.8 cycles/°; σ = 0.1 × eccentricity) and temporal frequency of 5–6 Hz, yielding velocities of 5.55–6.66°/s, respectively) were selected to match the approximate sensitivity of MT neurons (Bair and Movshon, 2004).

Each motion stimulus presentation consisted of seven consecutive motion pulses lasting 150 ms each (9 frames on the 60 Hz display; 18 frames on the 120 Hz display), producing a motion sequence of 1050 ms in total duration. On any given pulse, a number of Gabor elements would have their carrier sine waves drift in unison to produce motion (“signal elements”), and the remaining would counter-phase flicker (“noise elements”). Within any given pulse, signal elements were spatially assigned at random within the grid, and all signal elements drifted in the same direction.

Motion strength on pulse *i* was defined as the proportion of signal elements of the total number of elements, the value of which was drawn from a Gaussian distribution, *X_i_ N*(μ*_k_*,*s*) and rounded to the nearest integer, where *k* is the distribution index for the five trial types (strong left, weak left, zero-mean, weak right, strong right). Thus, while each pulse within a sequence could take on any value (and either sign/direction) from distribution *N*(μ*_k_*,*s*), the expectation of a sequence would be μ*_k_* (Fig. 1*b–d*). The subjects were rewarded for selecting the target consistent with the sign of the motion pulse sequence sum (i.e., the net direction), independent of the distribution μ*_k_* from which the pulses were drawn.

Subjects performed the motion discrimination task with three variations of temporal stimulus statistics (Levi et al., 2018). First was the flat-stimulus, in which expected motion strength was uniform over time within a trial. In other words, the mean of the motion strength distribution *N*(μ*_k_*,*s*) would be held constant throughout a stimulus presentation (i.e., the mean of the distribution from which *X_i_* was drawn was fixed at μ*_k_*), for pulses 1–7 (Fig. 1*b*).

Next, subjects encountered the late-stimulus, where motion strength was reduced substantially in early pulses, but not in late pulses (Fig. 1*c*). In this condition, μ*_k_* is set to 0 for the first pulse (*i* = 1), and reaches its expected value (μ*_k_*) by pulse 7. Finally, the opposite is done for the “early-stimulus” condition (Fig. 1*d*), in which the first pulses maintain mean motion strength equal to μ*_k_* and later pulses have a mean near zero. In the late and early-stimulus conditions, the transition from μ*_k_* at pulse 1 to μ*_k_* at pulse 7 is governed by a logistic function with parameters chosen to result in a smooth transition between the first three and last three pulses (midpoint = four pulses; slope = 0.3).

All subjects began the experiments with the flat-stimulus condition (Monkey L, 13 sessions; Monkey N, 10 sessions). After multiple sessions of stable psychophysical performance, the stimulus was changed to the late-stimulus conditions (Monkey L, 11 sessions; Monkey N, 11 sessions). Finally, after multiple sessions of stable psychophysical performance the stimulus was changed to the early-stimulus condition (Monkey L, 11 sessions; Monkey N, 15 sessions). Subjects were exposed to only one stimulus condition per session and were not cued as to which stimulus condition they were viewing before or during any given session (other than the stimulus statistics themselves).

Throughout all conditions, there existed a subset of “zero mean” trials in which μ*_k_* = 0 for all seven pulses, regardless of whether the stimulus condition is flat, late, or early. Sessions also contained 5–10% frozen seed trials, which were identical stimulus displays. The “frozen noise” stimulus always summed to zero, had the same temporal structure across sessions, and was completely identical within sessions. Subjects were rewarded at random on frozen noise trials.

#### Electrophysiology.

We performed electrophysiological recordings from MT of two rhesus macaques, one male (age, 6 years) and one female (age, 11 years). A custom titanium chamber was fabricated and placed over the superior temporal sulcus and intraparietal sulcus to allow for a dorsal approach to access area MT. Chamber placement was as guided by structural MRI and cranial landmarks. Extracellular recordings were performed using linear electrode arrays from Plexon (U-Probe, V-Probe, or S-Probe; 24 or 32 channels; spacing, 50–100 μm).

MT was identified using electrode depths and paths (i.e., sulcal anatomy), and functional mapping. Functionally, MT was identified via the size and location of RFs, and the preponderance of direction-selective neurons. MT units were hand mapped using a field of moving dots with experimenter control of stimulus location, aperture size, dot speed, dot size, and dot density. Upon choosing the stimulus location that maximally drove the highest number of neurons, direction tuning was measured by 500 ms presentations of a randomly drawn direction of motion from 1 of 12 directions from 0° to 330°. A total of 71 recording sessions were performed: 23 during the flat-stimulus condition (Monkey L, 13 sessions; Monkey N, 10 sessions); 22 during the late-stimulus condition (Monkey L, 11 sessions; Monkey N, 11 sessions); and 26 during the early-stimulus condition (Monkey L, 11 sessions; Monkey N, 15 sessions). As would be expected given our use of multielectrode arrays and a dorsal approach to area MT, we often encountered samples of neurons with diverse sets of tuning preferences during a single experimental session. For each session, the motion discrimination task was performed along the axis that corresponded to the preferred direction of the maximum number of units.

Spike sorting was performed using KiloSort (Pachitariu et al., 2016) followed by manual merging and splitting of clusters as necessary. A total of 583 units were identified: 161 during the flat-stimulus condition; 219 during the late-stimulus condition; and 203 during the early-stimulus condition.

### Statistical analysis

#### Behavioral analysis.

Subject choices in the direction-discrimination task were analyzed with a maximum likelihood fit of a three-parameter logistic function (Wichmann and Hill, 2001), assuming a Bernoulli distribution of binary choices, in which the probability of a rightward choice is *p* and leftward choice is 1 − *p*, where *p* is given by the following:

$$p=\gamma +\left(1-2\gamma \right)\frac{1}{1+e^{-\beta (x-\alpha )}},$$

where *x* is the net motion strength value (*z* scored over all sessions for each subject separately), α is the bias parameter (reflecting the midpoint of the function in units of motion strength), β is the slope (i.e., sensitivity, in units of log-odds per motion strength), and γ captures the lapse rate as the offset from the 0 and 1 bounds. Error estimates on the parameters were obtained from the square root of the diagonal of the inverse Hessian (second derivative matrix) of the negative log-likelihood. The temporal weighting kernel (which we also refer to as “temporal weighting strategy” or “temporal weighting profile”) was computed using ridge regression via maximum likelihood. The log-posterior of the psychophysical weights is given by the following:

$$L(w)=\overset{N}{\underset{i=1}{\sum }}\left[Y_{i}w^{⊤}X_{i}-\text{log}\left(1+\text{exp}\left(w^{⊤}X_{i}\right)\right)\right]+\lambda ∥w{∥}^{2},$$

where *Y* ∈ {0,1} is the choice for a given trial and *X* is a vector of motion strength for the seven pulses in the trial, augmented by a column of ones (to capture bias). Motion strength is represented as the number of signal elements out of the total number of (signal plus noise) elements. The regularizer λ was estimated using evidence optimization (Sahani and Linden, 2003). Psychophysical weights are normalized by the Euclidean norm of the vector of weights. The seven temporal weights assigned to the seven motion pulses, *w*, were computed by using all trials within a session. These include trials, where μ*_k_* was set to zero (i.e., “zero-mean trials,” where motion on a given pulse is temporally independent of all other pulses in the sequence), and trials where μ*_k_* was set to a nonzero value (“signal trials,” where motion is correlated over pulses).

#### Logistic regression neural decoder.

To interrogate the roles and relationship of direction-related and decision-related signals, we used various decoding methods to approximate how information may be gleaned from groups of MT neurons. The first method we used was logistic regression directly between spike counts and the binary direction or choice on each trial (Kiani et al., 2014; Yates et al., 2020). The regression is done for each session such that each neuron is a feature in the model, where every neuron received a weight according to how well it predicts the binary outcome of interest. The result is a linear readout model that allows for maximal prediction of the stimulus direction or the choice of the animal.

Specifically, the decoding weights are calculated as coefficients in a logistic regression between trial spike counts (summed over a window starting at stimulus onset and ending 150 ms after stimulus offset) and one of two binary variables (the stimulus direction, or the observer's choice) using the MATLAB function glmfit. The choice decoder weights were calculated using only the zero-sum, frozen noise trials, while the direction decoder used all other trials.

The probability of the stimulus direction of a trial or the choice given the firing rate of each neuron is given by the following:

$$p(X|Y,\beta )=\frac{\text{exp}(Yb)}{1+\text{exp}(Yb)},$$

where *b* = β*_0_* + _∑_*^N^_i_*_=1_ β*_i_X*_i_ for *N* neurons present during a session. *X* is a vector of spike counts per neuron, and the choice or direction is *Y* ∈ {0, 1}. The weights are then applied to the temporally binned trial spike rates of their respective neuron. Spikes were counted in 10 ms bins and smoothed with a 50 ms boxcar. This was expressed in terms of rates by dividing by the bin size. The result is a population response that best represented stimulus or choice information present in a recording session.

The resulting decoder output was then used to calculate population-level choice probability (CP) for each session. We measured CP over the course of stimulus presentation as a metric of trial-by-trial correlation between neural activity and decisions, given a fixed stimulus. CP was calculated as the area under the receiver operating characteristic curve generated from choice-conditioned distributions of the reweighted activity in each temporal bin. CP time course traces were smoothed with a 100 ms boxcar for visualization.

#### Latent factor analysis.

To better understand how stimulus and perceptual choices are encoded across the population, we used the variational latent Gaussian process (vLGP) method (Zhao and Park, 2017) to extract single-trial, low-dimensional latent factors from population recordings in area MT. Conventional analysis methods such as traditional factor analysis or principal component analysis make incorrect assumptions for spiking activity (e.g., Gaussian distributed) or assume linear dynamics that lack the complexity to describe nontrivial computations. vLGP overcomes these disadvantages by imposing a general (nonlinear) Gaussian process prior on the latent factors and assuming a point-process observation model to account for spikes. The method is explained in more detail below.

Spike counts were binned at 10 ms, using the time between target onset and reward. We assumed that the spatial dimensions of latent factors are independent and imposed a Gaussian process before the temporal correlation of each dimension, where **x***_k_* denotes the *k*th dimension of the latent factors, as follows:

$$x_{k}∼N(0,K).$$

To obtain smoothness, we used the squared exponential covariance function and respective covariance matrix **K** in the case of discrete time. Let *y_tn_* denote the occurrence of a spike of the *n*th neuron at time *t*, *y_tn_* = 1 if there was a spike at time *t*, and *y_tn_* = 0 otherwise. **y***_t_* is a vector of length *N*, the total number of neurons in a session, that concatenates all neurons at time *t*. The spikes **y***_t_* are assumed to be a point-process generated by the latent state **x***_t_* at that time via a linear–nonlinear model, as follows:

$$y_{t}∼\text{Poisson(exp}(Ax_{t}+b)).$$

To infer the latent factors (**x***_t_* for each trial) and the model parameters (**A** and **b**), we used a variational inference technique, as the pair of prior and likelihood do not have a tractable posterior. We assumed parametric variational posterior distribution of the latent factors, as follows:

$$q(x_{k})=N(\mu_{k},\Sigma_{k}).$$

We analyze the mean {**μ***_k_*} as the latent factors in this study. The dimensionality of the latent factors was determined to be four by leave-one-neuron-out cross-validation on the session with the largest population. All the sessions with at least four simultaneously recorded units were included in this analysis (Monkey N, 13 sessions; Monkey L, 28 sessions).

#### Pulse-triggered average.

To measure the relationship between the time-varying pulse strength and the inferred latent factors, we measured the contribution of pulses to the latent factors. The pulse-triggered average (PTA) measures the change in latent factors resulting from an additional pulse at a particular time of unit strength. To calculate the PTA, we used the pulse stimulus and latent response at 1 ms resolution. For each session, let *s_i_* denote the value of the *i*th motion stimulus, and let *x_tk_* denote the *k*th dimension of the latent factors at time *t*. All trials were concatenated such that the latent factors **X** is a matrix of length, *T* × 4, where *T* is the total time. For the *i*th pulse, *s_i_* is the number of Gabor elements pulsing, with *s_i_* > 0 for pulses in one direction and *s_i_* < 0 for pulses in the other direction. To calculate the temporal lags of the PTA, we built design matrices, **D** = [**D**_1_, **D**_2_, …, **D**_7_]. For the *i*th pulse, the design matrix **D***_i_* is a *T* × 28 matrix that consists of four cosine basis functions at the 4*i* + 1, 4*i* + 2, …, 4*i* + 4th columns and 0 elsewhere. These basis functions start at 0, 50, 100, and 150 ms after the onset, last 100 ms each, and span the rows of **D***_i_*. The magnitude of the bases is equal to the corresponding pulse value *s_i_*. We calculated a separate **D***_i_* value for each of the seven pulses, concatenated them to obtain a design matrix for all seven pulses, and estimated the weights with L2 regularization, as follows:

$$X=DW+EW=\text{argmin}∥X-DW{∥}_{2}^{2}+\lambda \prime ∥W{∥}^{2},$$

where **W** is the weight matrix to estimate, **E** is the Gaussian noise matrix, and the regularization hyperparameter λ′ was chosen by the generalized cross-validation (GCV; Golub et al., 1979). The estimation was performed using scikit-learn (Pedregosa et al., 2011). The PTA was calculated with the design matrices of unit-strength pulse and the estimated weights **W**. We smoothed the PTA with a temporal Gaussian kernel (width, 40 ms). Individual session PTAs are normalized by the peak response to highlight temporal dynamics within the stimulus period.

Subject to arbitrary rotations, a latent trajectory forms an equivalence class of which the members have the same explanatory power in the vLGP model. We seek a particular rotation for each session that makes the encoded task signal concentrate in the first few dimensions. By singular value decomposition, **W**^⊤^ = **USV**^⊤^, we rotate the factors **x** to **U^⊤^x**. The regression weights are thus sorted by the task signal explanatory power, as are the factors.

#### Choice decoder.

Since there were some recording sessions with a less than ideal number of frozen trials (identical visual motion trials), we used the “weak” trials where the discrimination accuracy of the monkeys was <65% to calculate choice probability. We started at the trials of zero pulse coherence and gradually increased the magnitude of coherence (absolute value) until the correct rate reached the threshold. One session contained <100 weak trials and was excluded from this analysis. Note that weak trials were included only for vLGP analyses, while the logistic regression decoder (which was in strong accordance with vLGP) used only zero-sum, frozen noise trials.

We removed the stimulus information that is encoded in the latent factors of weak trials by regressing out the pulses and analyzing the residuals. The latent factors were rebinned at 100 ms resolution where the value of each bin is the sum of latent state **x***_t_* or spike counts **y***_t_* over the bin for *t* = 1,2,…,*T*. For each *t*, we assumed the following linear model:

$$x_{t}=\overset{7}{\underset{i=1}{\sum }}w_{ti}s_{i}+e,$$

where *s_i_* denotes the strength of the *i*th pulse, **w***_ti_* is the weight vector corresponding to the bin and pulse, and **e** is the homogeneous Gaussian noise across all bins. We estimated the weight vector by least-squares with L2 regularization to prevent overfitting, as follows:

$$w_{ti}=\underset{w_{ti}}{\text{argmin}}∥x_{t}-\overset{7}{\underset{i=1}{\sum }}w_{ti}s_{i}{∥}_{2}^{2}+\tilde{\lambda }∥w_{ti}{∥}_{2}^{2}.$$

Again, the regularization hyperparameter $\tilde{\lambda }$ was chosen by GCV. We then analyzed the contribution of behavioral choice on the residuals, as follows:

$$r_{t}=x_{t}-\overset{7}{\underset{i=1}{\sum }}w_{ti}s_{i}.$$

For the whole trial, we used the sum residual of the windows *r* = ∑*_t_* **r***_t_*. The range of *t* depends on the period of interest. We trained logistic models, which we referred to as choice decoders, to predict the choice on each trial using latent factors. The weights **β** and bias β_0_ were estimated by maximum likelihood with L2 regularization, as follows:

$$\beta ,\beta_{0}=\underset{\beta ,\beta 0}{\text{argmax}}\text{logL}(\text{choice}|r;\beta ,\beta_{0})-\hat{\lambda }∥\beta ,\beta_{0}{∥}_{2}^{2}.$$

The regularization hyperparameter $\hat{\lambda }$ was chosen via fivefold (balanced classes in test set) cross-validation for every session individually. The estimation was performed using scikit-learn.

#### Choice mapping.

The conventional choice probability applies only to univariate variables. However, the latent factors and population activity are multivariate. We transformed the multivariate variables mentioned above onto a one-dimensional subspace that has the same direction as the choice through the choice decoders, as follows:

$$c=\frac{1}{1+e^{-\beta^{⊤}r-\beta_{0}}}.$$

We refer to this transform as the “choice mapping.” The quantity *c* is a normalized value within [0, 1] that maps the residual onto the choice direction (Lueckmann et al., 2018) and enables pooling across sessions.

To prevent potential inflation of choice probability because of multidimensionality (3D), we regularized the choice decoder and used only the choice mapping on the test set (pooled samples held out by cross-validation). This approach guarantees that choice probability will not be overestimated.

We pooled these mappings across all sessions. Using different subsets of latent factors as *r* in the mapping, we obtained the choice mapping of the stimulus and nonstimulus dimensions of the latent factors. Then we calculated the choice probability of the corresponding dimensions based on the values. To investigate the time course of choice probabilities, we performed choice mapping on the whole dataset with nonoverlapping moving windows. For the fixed readout analysis, we estimated the weights using the mean value of 0–1.2 s from stimulus onset for the stimulus period, and −0.5 to 0 s from the saccade for the delay period. We use the weights to obtain readout and CP values with a 10 ms moving window and smoothed the CP values with a 100 ms boxcar. Finally, for dynamic readout, we estimated the weights and calculated the CP values within 100 ms moving windows individually.

## Results

We measured the time course of sensory-correlated and choice-correlated responses from simultaneously recorded groups of MT neurons using linear and nonlinear decoding approaches while rhesus monkeys performed a motion direction discrimination task. We manipulated the time course of stimulus evidence and confirmed that the subjects shifted their temporal weighting strategy to rely preferentially on the stronger periods of stimulus motion. We began recordings in each subject with a baseline “flat” stimulus phase for several experimental sessions, in which stimuli had a constant average motion strength over time within a trial, as is the case in most related experiments (Gold and Shadlen, 2007). We then shifted to several sessions in a “late” regime, in which the stronger motion was present in the second half of the stimulus. Finally, we performed several sessions in an “early” regime, in which the stronger motion was present in the first half.

### Observers change temporal weighting strategies according to stimulus statistics

Two trained rhesus macaques viewed sequences of seven motion pulses and indicated perceived net motion with a saccade to one of two response targets (Fig. 1*a*). We measured traditional psychometric performance (i.e., accuracy as a function of net motion strength on each trial), as well as the time course of weighting within each trial (i.e., the “psychophysical kernel,” which was estimated using logistic regression between motion strength at each pulse, and the binary choices; see Materials and Methods). We refer to the resulting set of regression coefficients, or weights, as the temporal weighting strategy.

**Figure 2.**
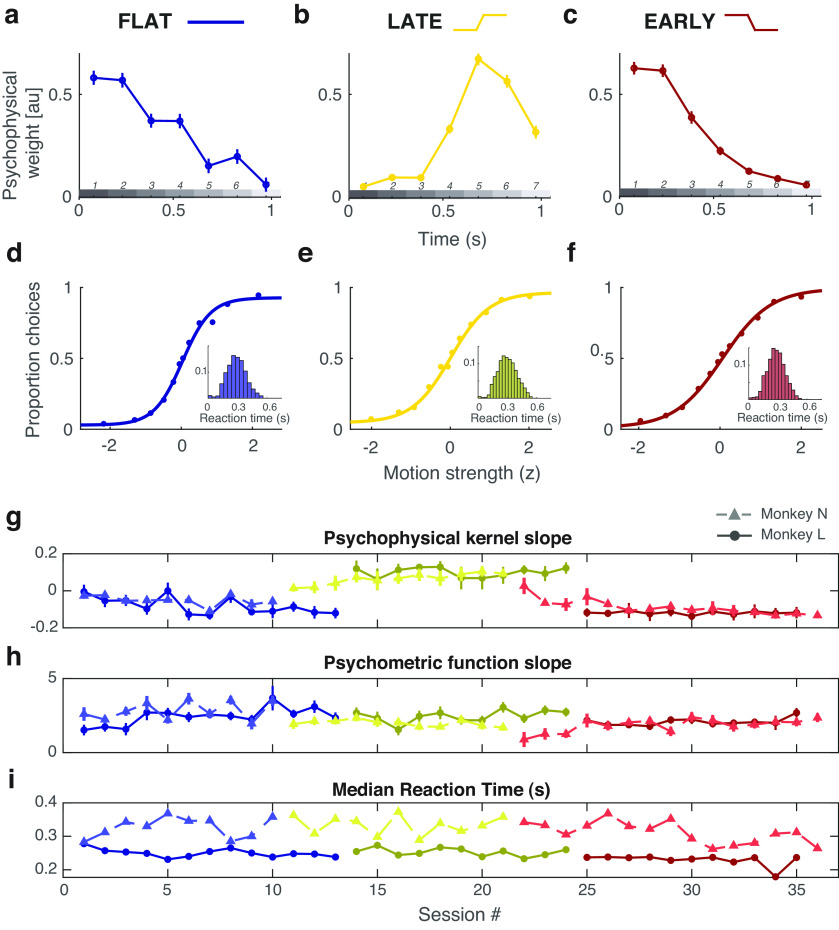
Temporal weighting strategy changes with stimulus statistics. ***a–c***, Temporal weighting behavior across conditions. ***a***, Subjects preferentially weight the early pulses despite uniform motion expectation over time. ***b***, Temporal weighting shifts during the late-stimulus condition to preferentially weight late pulses. ***c***, Behavior reverts back to early weighting when the stimulus statistics are biased toward high motion strength early. ***d–f***, Psychometric functions indicate that decisions varied with the direction and strength of motion, as expected. Insets show distributions of reaction times. ***g–i***, All metrics are largely consistent across sessions within a condition.

The motion discrimination task was performed in three contexts to manipulate the time course of behavioral weighting (Levi et al., 2018). First, in the flat-stimulus condition (Fig. 1*b*), the average motion over time was equal within a trial. Given that many traditional sensory decision-making studies use stimuli with uniform information over time, this flat-stimulus condition served as conventional baseline and reference conditions for our experiments. Subjects' temporal weighting strategies were biased to have higher weight on early stimulus periods, despite uniform motion expectation over time (Fig. 2*a*). This default early weighting strategy is consistent with many other findings (Huk and Shadlen, 2005; Kiani et al., 2008; Nienborg and Cumming, 2009; Yates et al., 2017; Kawaguchi et al., 2018) and likely reflects a combination (Okazawa et al., 2018; Levi and Huk, 2020) of improved sensory encoding at stimulus onset (Osborne et al., 2004; Churchland et al., 2010), and the consequences of early termination of the decision process, because of mechanisms like bounded accumulation (Kiani et al., 2008).

Next, we performed a series of experimental sessions in which the stimulus statistics were manipulated such that the average motion strength was high for the last three pulses, while the first three pulses were near zero. We refer to this as the late-stimulus condition (Fig. 1*c*). Although the first three pulses had motion strength near zero on average (regardless of full-trial net motion strength), it is important to appreciate that on individual trials there was still variable nonzero motion possible for any pulse (and hence, still provided useful information for decision-making). Subjects were rewarded based on the actual net motion direction presented on that particular trial, as opposed to the average or expected value based on the condition from which the trial was generated. This produced robust behavioral changes that tracked motion expectation in the stimulus design, such that weight on the first three pulses decreased substantially, and the highest psychophysical weight was placed on the later pulses (Fig. 2*b*).

Finally, we performed a series of sessions in which the stimulus statistics were changed such that the average motion strength was now high in the early half of the stimulus and was near zero for the last half of the stimulus; we refer to this as the early-stimulus condition (Fig. 1*d*). This successfully changed temporal weighting behavior back to pronounced early weighting, in which the first pulses received drastically higher weight than the remainder of the stimulus (Fig. 2*c*), in a manner overall similar to the default strategy during the flat-stimulus [flat: –0.091 (95% CI, –0.113, 0.069); late: 0.083 (95% CI, 0.015, 0.151); early: –0.109 (95% CI, –0.136, –0.081); slope of linear fit to the psychophysical kernel].

Importantly, the changes in temporal weighting occurred alongside otherwise stable psychometric performance. This confirms that temporal weighting changes were indeed a targeted shift in strategy and not merely a result of the more general effects of learning. Stimulus sensitivity could vary session to session, but did not show general learning effects over time, which would manifest as higher sensitivity as sessions progress, regardless of stimulus condition (Fig. 2*h*). In fact, psychometric sensitivity decreased slightly as the experimental conditions went on [Fig. 2*d–f*; flat: 2.5208 (95% CI, 2.3616, 2.6800); late: 2.0421 (95% CI, 1.9183, 2.1659); early: 1.6961 (95% CI, 1.5862, 1.8060); slope of psychometric function]. Likewise, while there was some variation in median reaction times (RTs) across sessions, it varied only slightly between the conditions at large (Fig. 2*i*; flat: 95% CI, 0.2880 ± 0.0011 s; late: 95% CI, 0.2960 ± 0.0011 s; early: 95% CI, 0.2810, ± 0.0010 s), and did not get increasingly faster as sessions progressed, as one might expect from the general effects of learning.

In summary, the temporal weighting strategy shifted in concert with the time course of expected motion strength, placing higher weight on portions of the stimulus when higher motion strength was expected based on the experimental phase. This confirms that our manipulation of stimulus statistics affected the time course of psychophysical weighting, allowing us to interpret the timescale of neural responses relative to the subject's readout of MT for motion discrimination, and to rely on an experimental manipulation that shifted this time course systematically across conditions.

### Changes in sensory encoding run opposite changes in temporal weighting strategy

**Figure 3.**
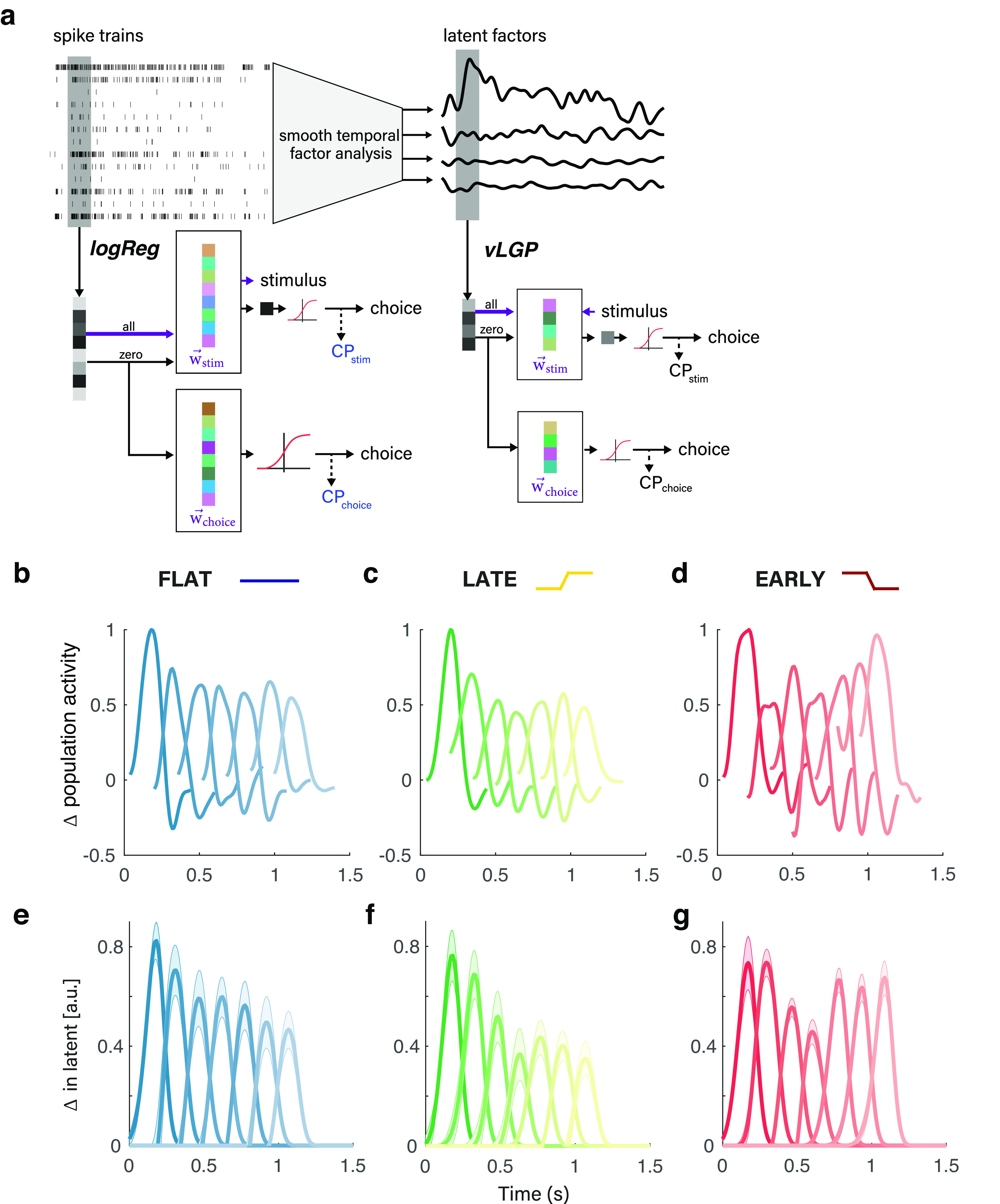
Time course of motion-driven MT response changes opposite that of changes in temporal weighting strategy. ***a***, From the simultaneously recorded spike trains, we found the linear projection that can best predict the stimulus direction (^→^*w*_stim_) or the choice (^→^*w*_choice_). To enhance the signal-to-noise ratio, we extracted low-dimensional latent factors that explain the correlations in the population spike trains using smoothing factor analysis (right). The first projection is found by the singular dimension explaining the stimulus drive for all trials (^→^*w*_stim_). The second is the choice information extracted from the top four latent factors altogether (^→^*w*_choice_). Projection of the frozen noise trials is still multidimensional and required further logistic regression to best predict the choice, defining the projection ^→^*w*_stim_ and corresponding CP_choice_. ***b–g***, The PTA describes the modulation of the stimulus axis by a pulse of unit motion strength for each of the seven pulses in the visual motion stimulus. ***b*–*d***, Example sessions showing modulation of the sensory response during the three stimulus conditions, where the PTA is calculated on the one-dimensional, reweighted population response that is the output of the logReg decoder. ***e*–*g***, Average PTAs across all sessions for each condition using the vLGP decoder. Error bars reflect 68% CIs (±1 SEM).

We recorded ensembles of single and multiunit activity from area MT while monkeys performed the direction discrimination task across the manipulation of the temporal weighting strategy described in the previous section. We used both linear and nonlinear ensemble decoding frameworks to extract information about visual motion direction (i.e., stimulus) and decisions (i.e., choice) from groups of simultaneously recorded MT neurons (Fig. 3*a*). As a simple starting point, we used logistic regression (“logReg”) between the raw trial spike count vectors and either the stimulus direction (the “direction” axis) or the psychophysical choice (the “choice” axis) to find a reweighted population response that best mapped neural activity to the binary stimulus or choice (Fig. 3*a*, left). Such linear models are likely easy for the brain to implement, but are limited in how they can capture relations between neurons and between neural activity and experimental factors. We therefore also used a more advanced nonlinear dimensionality reduction technique (vLGP model) to extract smooth low-dimensional latent factors that explain correlations within the population spike trains (Zhao and Park, 2017; Zhao et al., 2020; Fig. 3*a*, right). This provides the ability to more effectively capture the complex joint statistics of the neural population by leveraging the low-dimensional assumption and effectively denoises the spiking activity. Decoding based on vLGP also uses logistic regression to map the intermediate representation of ensemble activity (i.e., the inferred latent factors) to the stimulus or the choice.

Surprisingly, we observed large changes to the time-varying sensory response of the MT that were incommensurate with perceptual readout. To describe the time-varying gain on motion throughout each trial, we calculated the pulse-triggered average, which characterizes the change in the neural response to unit-strength motion (i.e., a single Gabor element drifting in one direction) relative to the onset of each of the seven pulse epochs (for details, see Materials and Methods). As the temporal weighting strategy shifted across conditions, one might expect nothing to change in MT, consistent with a constant (and thus largely veridical) representation of visual information despite changes in readout/weighting strategy. An alternative hypothesis, based on temporal attention, would predict gain modulation congruent with behaviorally upweighted and downweighted stimulus epochs (Ghose and Maunsell, 2002). Instead, to our surprise, we observed changes to sensory encoding with an unintuitive, if almost paradoxical, link to psychophysical direction discrimination.

In the flat-stimulus condition, there was a modest decrease in the sensory response over time (i.e., PTA magnitude fell across the seven pulse epochs; Fig. 3*b*,*e*). Such a gradually declining time course is consistent with known adaptation phenomena in many visual brain areas and has been observed in MT during viewing of this same stimulus (Yates et al., 2017). However, during the late-stimulus condition, the average time course of the sensory response was not distinguishable from that of the flat-stimulus [exponential fit, *a* · exp(*b* · *x*); flat: *a* = 0.86 (95% CI, 0.77, 0.96); *b* = −0.09 (95% CI, −0.12, −0.06); late: *a* = 0.86 (95% CI, 0.67, 1.06); *b* = −0.15 (95% CI, −0.21, −0.08); Fig. 3*c*,*f*]. Most importantly, this gradual decrease over the seven pulses is precisely the opposite of the behavioral profile, which shows relative downweighting of early pulses and upweighting of later pulses. And, most strikingly, when subjects switched to the early-stimulus condition, the sensory response showed a stark upweighting of later pulses, resulting in a dramatically nonmonotonic, U-shaped profile (Fig. 3*d*,*e*). While the flat and late conditions were well described by an exponential model (flat, *R*^2^ = 0.93; late, *R*^2^ = 0.87), the drastic change in shape means that the early condition was not (early, *R*^2^ = 0.05). Indeed, the early condition was the only time when responsivity significantly increased as time progressed [maximum (±SE): pulse 4, 0.46 (0.41 ± 0.51); to pulse 5, 0.67 (0.62 ± 0.71)]. Likewise, the responses to pulses 5, 6, and 7 were significantly greater than those of both the flat and early conditions [flat: pulse 5, 0.56 (1 SEM, 0.46 6 0.66); pulse 6, 0.49 (1 SEM, 0.39 6 0.59); pulse 7, 0.46 (1 SEM 0.39 6 0.54); early: pulse 5, 0.67 (1 SEM, 0.62 6 0.71); pulse 6, 0.64 (1 SEM, 0.58 6 0.69); pulse 7, 0.68 (1 SEM, 0.61 6 0.74); mean pulse maximum]. This is unexpected because of the relative lack of average stimulus strength in the final three pulses during the early condition compared with the flat and late conditions. Once again, this is also directly at odds with the temporal weighting of behavior, which sharply favors the first two to three pulses over the rest. This modulation is counterintuitive from standard perspectives, which would predict that if any changes in sensory response are evident, they would be reflected by increases in response to stimulus portions that were weighted more strongly for decision-making.

Instead of gain changes that reflect behavioral readout strategy, the sensory response modulations we observed make more sense viewed as compensating for the “missing” signal relative to a time-stationary motion expectation. In our experiments, both animals were trained extensively on the flat condition before undergoing temporal manipulation. The change in gain thus manifested as a function of the mismatch between this apparently “default” temporally uniform expectation of motion and the statistics of the currently encountered condition. In more detail, during the late condition motion strength was decreased in the early portions of the stimulus, but the PTA did not reveal a decrease in the response to those pulses (Fig. 3*c*,*f*). In fact, the only noteworthy difference from the flat to the late condition was a decrease in the response to pulse 4, which can be interpreted as a faster falloff of the floor responsivity of the population, just before the highest behaviorally weighted pulses [pulse 4: flat mean peaks (±SE), 0.60 (0.52, 0.68); late mean peaks, 0.37 (0.26 0.47)]. During the early condition, the motion strength on later pulses was decreased, but the PTA revealed a striking gain increase on these portions of the stimulus for which the expected motion was quite weak (Fig. 3*d*,*g*). Thus, while the temporal weighting evident in behavior changed across conditions in a way that tracked changes in stimulus statistics (i.e., weighting the stronger periods of motion more, and the weaker periods of motion less), the response of MT to motion was changed inversely to those patterns.

Consistent with prior work (Zhao et al., 2020), the motion stimulus was primarily present in one dimension within the space of the vLGP latent factors, termed the stimulus axis. In fact, we found that the sensitivity of a neuron to task-relevant motion directions (*d*′) was highly correlated with its response in the stimulus axis (*r* = 0.484), but not with the null space (three dimensions, *r* = 0.106). The vLGP-based PTA values (Fig. 3*e–g*) were estimated on the stimulus axis. Thus, the correlation between the *d*′ value of a neuron and the stimulus axis (but not the other latent factors) confirms that, despite counterintuitive changes to the time course of the PTA, it is still directly and proportionally informed by the neurons most sensitive to the task.

### Choice-correlated activity in MT is large but does not align with stimulus encoding or behavioral readout

We observed the presence of substantial choice-correlated activity in the MT population response (evident via both logReg and vLGP), achieving large peak magnitudes (>0.6), as measured by CP; although we use CP as a conventional metric in this article, we emphasize that by calculating it on various dimensions of the ensemble response, we have generalized it beyond the classical approach of only looking at choice-correlated activity defined along the stimulus axis for one neuron at a time (Britten et al., 1996).

**Figure 4.**
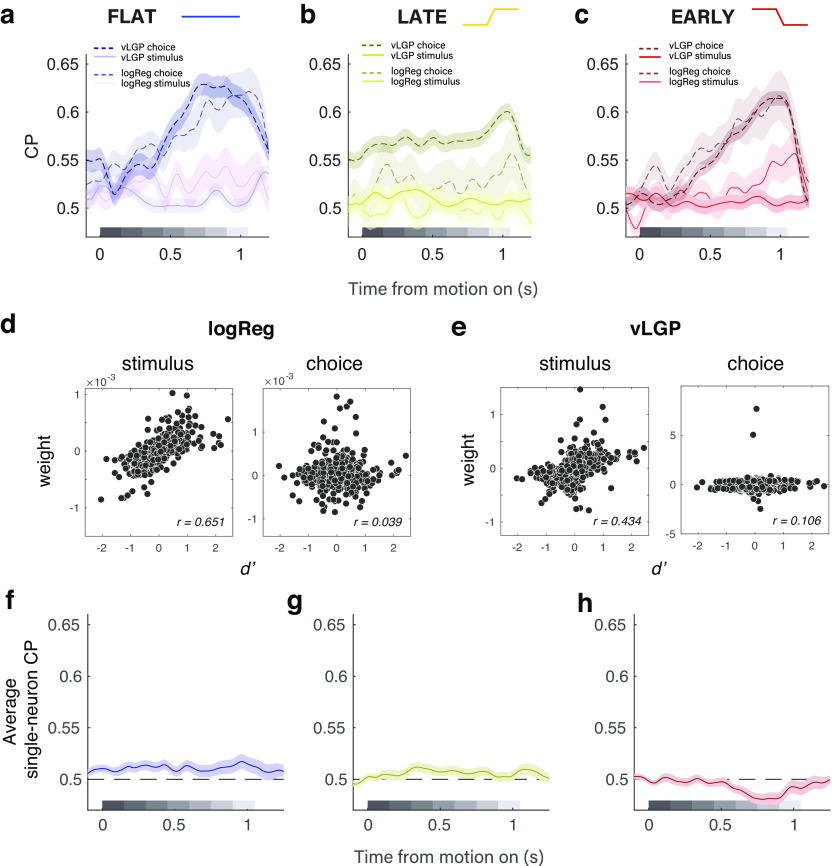
Both linear and nonlinear ensemble analysis approaches reveal strong choice-correlated activity in MT distinct from motion encoding or psychophysical readout of motion signals. ***a–c***, Time course of population-decoded choice probability during flat (***a***), late (***b***), and early (***c***) conditions. Solid versus dashed lines denote stimulus versus choice dimensions, respectively. Darker traces in the foreground denote CP calculated from latent factors, while semi-transparent traces denote logistic regression-based CP. Choice probability was consistently highest late during the stimulus period, regardless of the changes in temporal weighting strategy across conditions. ***d***, Individual neuron weights in the logReg model to predict direction (left) and choice (right) as a function of task-relevant direction sensitivity (*d*′). The *d*′ value of a neuron was strongly correlated with its weight on the direction axis (*r* = 0.651) and uncorrelated with its weight on the choice axis (*r* = 0.039). ***e***, The *d*′ value of a neuron was strongly correlated with the vLGP loading weights for the stimulus dimension (*r* = 0.484), and was only weakly correlated with the loading weights for the choice dimensions (*r* = 0.106). ***f–h***, Average time course of single-unit CP is noisier and smaller in magnitude than CP from the population decoders.

The largest choice-correlated activity was present in the population activity in a manner distinct from how the stimulus drove the ensemble of MT neurons. Via logReg, this was evident in significantly larger CPs along the choice axis over the direction axis (Fig. 4*a–c*), stemming from a weak correspondence between the weight of a neuron in one model compared with the other (*r* = 0.146), given that the sensitivity of a neuron to task-relevant motion (*d′*) was strongly correlated with its weight along the direction axis (Fig. 4*d*, left; *r* = 0.651), but not at all correlated with its weight along the choice axis (Fig. 4*d*, right; *r* = 0.039). The vLGP analysis showed that stimulus encoding was well described by a single dimension (the stimulus axis), but the stimulus axis had little choice information (rarely exceeding 0.5) when compared with the combined choice information in the top four latent factors altogether (Zhao et al., 2020; Fig. 4*a–c*). This is evident further in the correlation between the *d′* value of an individual neuron and the loading weight for the stimulus dimension (Fig. 4*e*, left; *r* = 0.434) and the lack of a correlation between the *d′* value and the combined weight in the remaining three nonstimulus, or choice, dimensions (Fig. 4*e*, right; *r* = 0.106). The value of the ensemble-level decoding approaches (implemented via logReg and/or vLGP) is highlighted by the fact that conventional, single neuron-based CP time courses are noisier and smaller in magnitude, with only small deflections from chance (Fig. 4*f–h*). Averaging over all neurons obscures the correlations between activity and choices that are revealed by the weighting schemes of the two population decoders.

Importantly, both analysis methods revealed that across pronounced, experimenter-induced, changes in temporal weighting strategy, the time course of choice-correlated activities never mirrored the time course of psychophysical readout (Fig. 4*a–c*). Instead, choice-correlated activity was consistently highest after the stimulus periods that were weighted the highest in the behavior. In the flat condition, both analysis approaches demonstrated increased choice probability during the last half of the stimulus, despite early weighting in the behavior. In the late condition, when behavior exhibited the strongest dependence on later portions of the stimulus, the strongest choice-correlated activity was still distinct from the stimulus-driven activity, and exhibited a more muted and flatter time course, though still characterized by an even later peak relative to the flat-stimulus condition. Finally, when subjects returned to an early weighting strategy in the early stimulus condition, the time course of choice probability returned to a similar rising profile, as originally measured during the flat condition. These observations are inconsistent with classical interpretations that choice probabilities reflect the feedforward consequences of sensory noise being read out as information about the stimulus because the bulk of the choice-correlated activity arose after the psychophysical readout of MT occurred. But, the form of the choice-related activity also challenges more recent interpretations that choice probabilities reflect feedback (Nienborg and Cumming, 2009; Wimmer et al., 2015; Cumming and Nienborg, 2016; Goris et al., 2017; Bondy et al., 2018; Zhao et al., 2020), because differential MT responses correlated with choice were not strongly aligned with the motion responses that gave rise to those decisions, and thus are difficult to interpret as a choice signal being “sent back” to the sensory neurons with direction preferences consistent with the decision. Rather, the sensory and nonsensory signals appear to be multiplexed at the population level in a way that suggests they are not likely to interact in a straightforward feedforward or feedback manner, if at all (Yu and Gu, 2018; Zhao et al., 2020; Quinn et al., 2021).

### Large choice-correlated activity also exists in the absence of the motion stimulus

**Figure 5.**
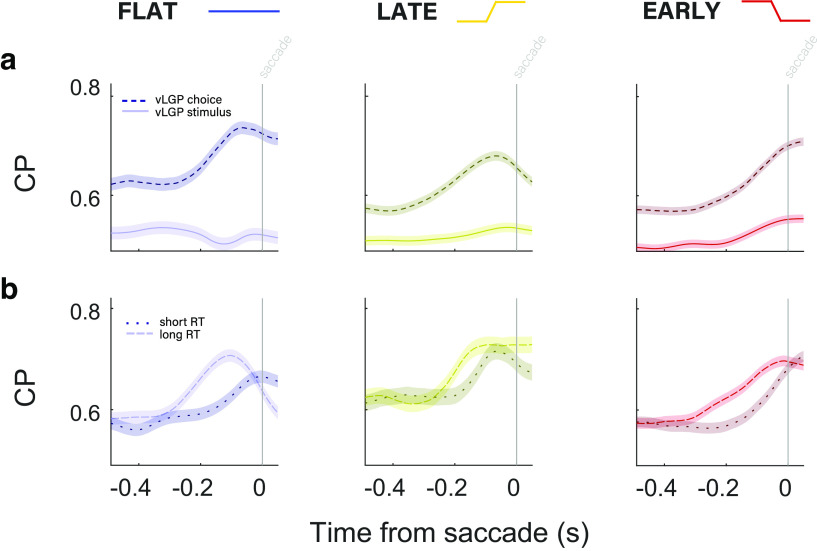
Presence of large choice-related signals in MT during poststimulus delay period. ***a***, CP along the choice (dashed lines) and direction (solid lines) axes, aligned to the time of the saccade. In all three conditions, there is high CP in the choice axis for the entire 500 ms preceding the saccade, without any stimulus drive. CP increased over the last 200 ms leading up to the saccade. There was relatively little CP along the stimulus axis. ***b***, Saccade-aligned CP along the choice axis only, separated by median RT. CP for longer RT trials (dashed lines) increased earlier than that of shorter RT trials (dotted lines). This was true in all three conditions.

We also observed another choice-related signal in MT of substantial magnitude. The vLGP analysis revealed significant choice-correlated activity after the offset of the motion stimulus, in anticipation of an upcoming saccade. There was a minimum 500 ms window between the stimulus offset and the disappearance of the fixation point that signaled the monkey could move their eyes to make their choice, and during this window we saw choice probabilities >0.7 (Fig. 5*a*). To be sure that this presaccadic CP was not contaminated by small eye movements within the fixation window, we calculated the speed of eye movements projected along the target–response vector in the same 500 ms window. We found only minuscule eye movements within this window, none of which showed any pattern with respect to time, or varied systematically with motion strength (median drift speed: strong trials, 0.024°/s; weak trials, 0.039°/s; zero-mean trials = 0.038°/s; ANOVA, *p* = 0.51). A similar lack of systematic effects of relative eye position (as opposed to velocity) was also noted.

The magnitude of poststimulus choice probability is comparable to, and often greater than, what we observed during the stimulus period, and is quite high compared with traditional measures of choice probability based on single-neuron measurements. Most importantly, the finding of large amounts of choice-correlated activity without the presence of a visual stimulus in MT strengthens the case for such signals being nonsensory in origin. The choice signal measured during the delay period is present when there is no sensory drive whatsoever, further ruling out interpretations of choice probabilities as a product of noise in sensory representations. Instead, its full magnitude (revealed by “looking off” the stimulus axis), late time course, and presence up to the response are more similar to the choice-related activity seen in a multitude of areas that are often considered much more cognitive or associative in nature, such as lateral intraparietal area and prefrontal cortex (Roitman and Shadlen, 2002; Mante et al., 2013).

Interestingly, the onset of CP during the delay period varied with RT in a way that suggests the choice signal is not simple premotor activity. If this were the case, we would expect that CP would increase later on trials with longer RTs compared with trials with shorter RTs. Instead, when reaction times were longer than the median RT, the saccade-aligned CP increased noticeably earlier than on trials with reaction times in the shorter half of the RT distribution (Fig. 5*b*). This was true of all three temporal stimulus conditions. The result is striking, especially given the fixed-stimulus experimental design and the coarse division of “short” and “long” RTs by median. Temporally divorced from stimulus processing and not tightly linked to motor behavior timing, this delay period choice signal appears to have a more cognitive origin reflecting the maintenance of choice information between stimulus and response.

### Time-varying readout of population activity confirms the dynamics of choice-related signals

**Figure 6.**
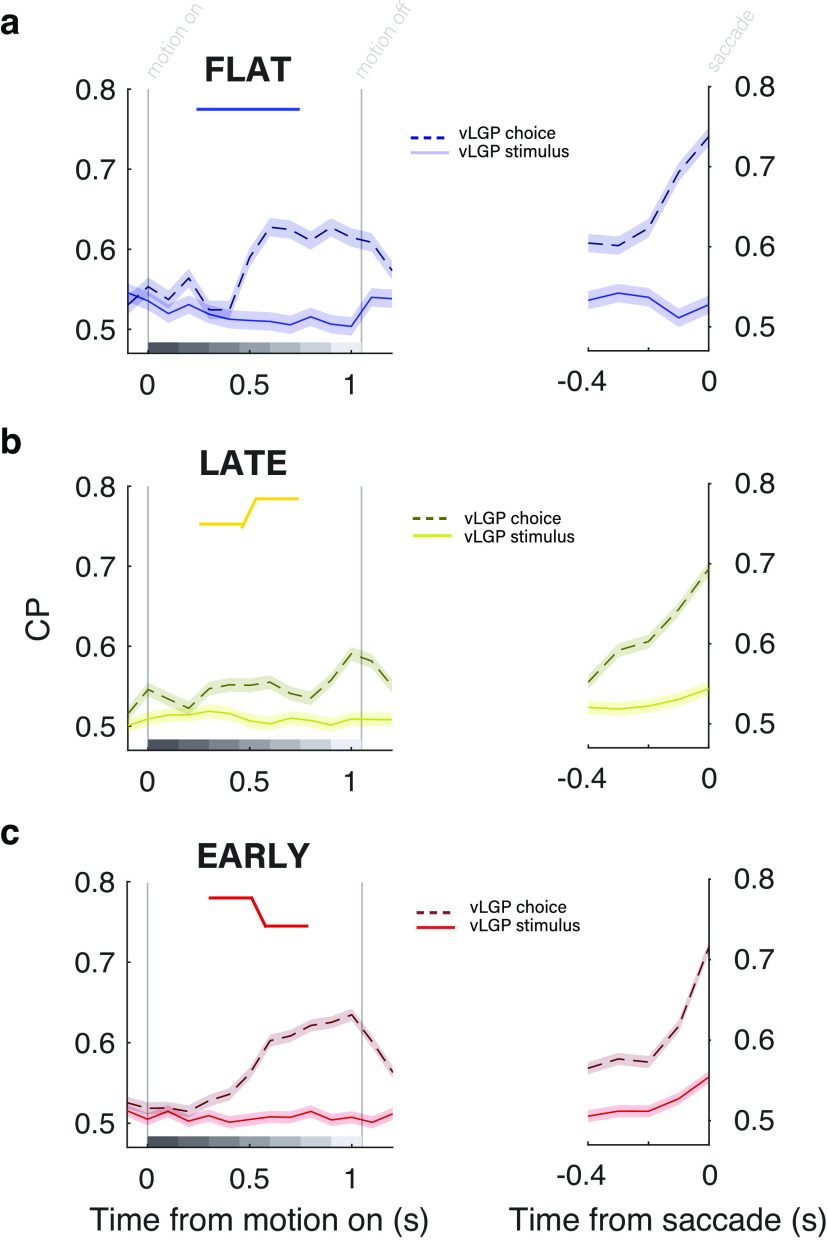
Time course of choice-related activity in MT area is similar when time-varying decoding weights are used. ***a–c***, Choice probabilities calculated with time-varying readout weights aligned to motion (left), and the saccades (right) for the flat (***a***), late (***b***), and early (***c***) conditions. CP along the choice axis is represented by dashed lines, while CP along the stimulus axis is represented by solid lines. Choice-axis CP was significantly higher in both the motion-aligned and saccade-aligned time frames. During motion (left), we confirmed that CP was highest during later stimulus epochs, after those with highest psychophysical weight. During the poststimulus period (right), we confirmed that CP increased primarily over the last 200 ms preceding the saccade to levels even higher than motion-aligned CP.

In all analyses leading up to this point, the weights used to decode the stimulus or the choice were calculated using the neural responses and/or the derived latent factors from the entire stimulus period. Even with this fixed temporal readout scheme, we saw nuanced temporal dynamics in both sensory-related and choice-related activity that differed from the time course of temporal weighting evident in the psychophysical behavior. Although, from a decoding perspective, using temporal fixed weights makes for a readout process that the brain might find easier to implement, we know very little about how sophisticated the decoding machinery of the brain might be (and indeed, our ability to manipulate the time course of motion weighting suggests that temporally static decoding is not a hard limit). Furthermore, from a purely statistical perspective, we were also motivated to consider decoding with a temporally dynamic readout scheme to confirm that the rich dynamics we observed were neither constrained nor distorted by the assumption of constant readout weights. We therefore performed further latent factor analyses in which weights were fitted and applied based on the activity within individual 100 ms bins for both the delay and motion periods (Fig. 6).

The time course of choice-correlated activity was quite similar from fixed to dynamic readout models. With temporally varied readout weights, the same pattern persisted: high CP late in the stimulus period regardless of temporal stimulus condition (Fig. 6, left). This is strong support for CP as a top-down signal that arrives in MT mostly after decisions have been made; that is, after the pulses with the highest weight in the psychophysical kernel. In this interpretation, during the late condition we have in essence delayed the decision and thus further delayed the decision-correlated activity that follows. The time-varying readout schemes also confirmed the dynamics in the poststimulus delay period. In all three conditions, CP was high throughout the delay period, but increased over the last 200 ms (Fig. 6, right). Along the stimulus axis, CP was flatter and closer to chance. Altogether, the similarity in CP time course between fixed and dynamic readout models suggests that a fixed weighting scheme is sufficient to describe the temporal patterns of choice information in MT during motion information both during and after the stimulus.

## Discussion

By manipulating the temporal weighting strategy of subjects while they performed a direction discrimination task, aided by ensemble recordings and population-level decoding analyses, we discovered multiple signals in MT that are distinct from its representation of motion direction. These findings expand our conception of MT, whose sensory representation of motion is solidly established to be used by later decision stages for perceptual reports and behavior. Striking changes in sensory response were associated with the mismatch between the current strength of sensory evidence and the learned time courses of sensory evidence. Although these large modulations affected the sensory encoding, they appear not to have affected the psychophysical behavior. Choice-correlated activity was also surprisingly strong, but was delayed relative to temporal weighting behavior, although the psychophysical temporal weighting strategy was under direct experimenter control. Furthermore, the choice-correlated activity was evident at the population level in a manner that was distinct from stimulus-driven responses in MT and was “readout irrelevant,” in that it was largest when the subjects were not primarily reading out the stimulus or even viewing a stimulus at all.

The changes we observed in sensory responses may seem paradoxical at first, as the gain was increased for periods of the stimulus during which the subjects applied the smallest amount of weight in forming decisions. This is opposite to the notion of attention affecting gain for parts of a stimulus that are more relevant for decisions (Treue and Maunsell, 1996; Seidemann and Newsome, 1999). But, these modulations appear more sensible when viewed as resulting from a mismatch between trained statistics and the current ones. The hyporesponsivity to late pulses in the late condition, and the hyper-responsivity to those same late pulses during the early condition, could both reflect a compensatory response to motion in the current condition compared with the expectation of the temporally uniform stimulus on which animals were trained. Indeed, potentially related homeostatic mechanisms have been observed in sensory cortex (Benucci et al., 2013). Through this lens, the temporal changes in the PTA reflect a recalibration of incoming information to meet the expectation of a temporally flat stimulus. Thus, even the sensory responses of MT are strongly affected by cognitive factors in ways that are dissociable from its well established—but no longer singular—role of representing retinal motion for the sake of perception and/or behavior.

Our findings regarding choice-related activity also add to the case for MT carrying substantial nonsensory signals. Having previously used ensemble recordings and population decoding to show that stimulus-related and choice-related activity in MT are distinguishable (Zhao et al., 2020), our findings in this study add several important facets. First, we exerted explicit control over the time course of psychophysical weighting, which allowed us to experimentally dissociate the psychophysical weighting from the time course of choice-correlated activity. By shifting the temporal weighting strategy, we effectively changed the average time of the decision, allowing us to confirm that choice signals followed primary decision formation when under explicit experimenter control. Second, we saw choice activity of substantial magnitude during the poststimulus delay period. This result rejects virtually any stimulus-based interpretation, as the choice signal was present when the sensory stimulus was not. These results also rule out straightforward forms of feedback creating choice-related activity, as those explanations require the decision-related feedback to be aligned with the sensory responses that gave rise to the corresponding choice. Furthermore, the delay period choice signal was not entirely explainable as premotor. Given all these distinctions, the parsimonious interpretation is that choice-related activity in MT is a distinct cognitive signal (or set of signals) that is best understood outside of the encoding of visual motion of MT. Although the presence of large choice-related but readout-irrelevant signals in macaque MT may be surprising at first, recent work in other species (also using ensemble recordings and analyses) has revealed widespread representations of choice-related and other task-correlated signals (Gründemann et al., 2018; Stringer et al., 2018; Musall et al., 2019).

### Implications of sequential experimental design

Because we used a sequential design in which we slowly changed across our three stimulus conditions, it is important to consider the possible effects of general learning that might have occurred gradually over time, and not as a result of our manipulation of stimulus statistics. We can rule out any general learning effects in our behavioral results, as we would expect these to manifest as higher sensitivity as sessions progress, regardless of stimulus condition. This was not true, as psychometric sensitivity decreased slightly as the experimental conditions went on (slope of psychometric function ± 95% CI: flat, 2.52 ± 0.15; late, 2.04 ± 0.12; early, 1.70 ± 0.11). Similarly, we can rule out such effects on the neural stimulus and choice signals. If changes in neural responses from the flat condition to the late condition were a result of generalized learning, we would expect those effects to continue on from the late condition to the early condition. This is not true of either the sensory or choice signals we saw in MT, in which the patterns were distinct to the stimulus condition and showed no general trends across the full set of experiments.

We used an experimental design in which we changed the temporal weighting condition after several sessions to examine how neurons adapted and learned in the periods when the observer adjusted from one temporal weighting strategy to a new one. However, we found ourselves unable to do so because the change in stimulus statistics was such a powerful manipulation that the monkeys shifted their strategies rather quickly, leaving us with little opportunity to examine what happens as they learned the new expected statistics. It would be interesting to know whether the results would change in any way given a different experimental order, or given a design that incorporates all stimulus conditions within experimental sessions. Indeed, future studies would be wise to incorporate random, short blocks in similar experiments to further rule out possible sequential effects; this would also allow for recording from the same groups of neurons during all experimental conditions.

### Population decoding approaches enable otherwise inaccessible insights

Our two analysis approaches revealed the temporal dynamics of the stimulus and choice responses of the MT that would not be as easily evident from classical, single neuron-based analyses. In the case of the response to motion, the average PTA of all recorded neurons showed trends reminiscent of those from our population decoders, but the temporal modulations were muted and could easily be overlooked and interpreted as stable across the three conditions. In the case of the choice signal, this was similarly evident in lower-magnitude CP and noisier time courses. We believe this is a relatively straightforward consequence of making simultaneous ensemble-scale recordings, as a raw average treats all neurons equally, regardless of their tuning to stimulus or choice, and would thus have detrimental effects on revealing signals.

This distinction between conventional metrics and population-level analyses highlights the value of the approaches used here. Indeed, even a simple weighted average, achieved via our logReg weights, was quite useful. It is important to appreciate that our samples of MT neurons within a session often contained a variety of tuning preferences, and that, while we ran the experiments along the directional axis that drives the greatest number of neurons according to their direction preference, there were often multiple cells with distinct tuning properties being fed into our decoders. By using logistic regression, we weighed each neuron according to its ability to predict the stimulus direction, and came away with an interpretable average that effectively downweighted the response of less relevant neurons. Although the brain may not be performing logistic regression per se, weighting the appropriate neurons for readout is a common assumption.

Likewise, not every neuron in MT, or in our samples of MT, is tuned for the choices of the animal (i.e., has high choice probability). In fact, we found that many neurons without strong direction selectivity had strong choice selectivity, and vice versa. Indeed, the choice response in MT appears to be a majority separate signal that lives nearly independent of its encoding of motion. We would have been blind to such relationships without the advantages of our current methods.

Thus, we view the logReg-weighted population response as a minimum necessary analysis for interpreting the nuances of high-dimensional recordings during complex behavior, and view vLGP as the more sophisticated approach that allows for deeper understanding of the population dynamics, which are made possible by leveraging assumptions of low-dimensional representations that explain the correlations within a high-dimensional space.

### Conclusions

These findings provide new connections between MT function and well established conceptual and empirical frameworks. The sensory modulations associated with mismatches between expected and observed time courses of motion align with both predictive coding and reinforcement learning models, both of which are abstractly based on errors between expected and encountered elements within a task (Rescorla and Wagner, 1972; Engel et al., 2015). Although our findings run opposite to the known effects of temporal attention (Ghose and Maunsell, 2002) or to attention-related gating of sensory responses (Seidemann et al., 1998), some work has decoupled attentional modulations in MT area and medial superior temporal (MST) area from task performance (Zénon and Krauzlis, 2012). Our dissociation between MT modulations and task performance may be related, although in our case, their dependence on the strategic history of the subjects revealed signals that are not wholly irrelevant to the task, but are just not related to the formation of decisions on a trial-by-trial basis. This opens up the possibility that some attention-like phenomena may arise from expectations of stimulus statistics, instead of being modulations of sensory data per se. The poststimulus choice signals we observed in MT may be related to prior observations of small-amplitude, but tuned, persistent activity in MT (Bisley et al., 2004). Our findings suggest that those initial observations of relatively small changes in spike rate may have simply caught a glimpse of larger nonsensory signals preceding the saccadic decisions mostly missed by single-unit recordings that cannot see alternate effects on population activity across diversely tuned neurons. Finally, related work using a motion categorization task has revealed strong nonsensory, category-related activity in area MST, but not area MT (Freedman and Assad, 2006; Zhou et al., 2020). Such category-related activity can also be thought of as “choice-correlated,” as distinct from purely sensory driven. Although the tasks, training histories, and analytic approaches differ between that work, our findings suggest that the apparent distinction between MT and MST areas regarding the presence of such category/choice activity might be less strict than previously observed. Again, the potential for ensemble recordings and corresponding analyses may have been critical for not just observing these nonsensory signals in MT, but for appreciating their substantial magnitude.

To conclude, our manipulation of temporal weighting strategy revealed a dissociation of sensory response gain that did not appear to play out in decisions about visual motion. Likewise, our approach of using ensemble recordings and population decoding allowed us to see large choice-related signals that were not just temporally dissociated from psychophysical weighting (or even stimulus viewing), but that were large in magnitude and distributed across the population in a manner distinct from how visual motion direction is represented. Together, these signals and modulations call for consideration of MT well beyond its role in the encoding of retinal motion and, more generally, challenge simple conceptions of sensory gain and decision-related feedback as being clearly task relevant. Understanding the population coding structure and functional roles of such task-related but nonsensory computations are new open questions.
